# Anchoring in the past, tweeting from the present: Cognitive bias in journalists’ word choices

**DOI:** 10.1371/journal.pone.0263730

**Published:** 2022-03-02

**Authors:** Jihye Lee, James T. Hamilton

**Affiliations:** Department of Communication, Stanford University, Stanford, California, United States of America; University of Hradec Kralove: Univerzita Hradec Kralove, CZECH REPUBLIC

## Abstract

This study examines journalists’ language in their reporting and what their word choices reveal about their cognitive mindsets. Reporters on the campaign trail often cannot afford to engage in systematic information processing as they distill complex political situations under deadline pressures. Twitter’s emphasis on speed and informal cultural milieu can further lead journalists to rely on heuristics and emotions. Drawing upon insights from theories of the mind, memory, and language, this study explores how cognitive biases are embodied in journalistic work across different media. We built a large-scale dataset of text corpora that consisted of more than 220,000 news articles, broadcast transcripts, and tweets generated over a year by 73 campaign reporters in the 2016 U.S. presidential election. Leveraging this unique dataset of journalistic outputs from a campaign season, we conducted automated text analyses. Results suggest that heuristics and intuitive thinking played a significant role in the generation of content on Twitter. Journalists infused their tweets with more emotion, compared to when they appeared in traditional media such as newspapers and broadcasts. Journalists’ tweets contained fewer words related to analytical and long-term thinking than their writing. Journalists also used informal language in their tweets to connect with their audiences in more personal and casual manners. Across all media examined in the study, journalists described the current race by drawing upon their experience of covering prior presidential elections, a form of anchoring heuristic. This study extends the use of cognitive biases in politics to a new realm, reporting, and shows how journalists’ use of language on the campaign trail reflects cognitive biases that arise when individuals make decisions under time pressure and uncertainty.

## Introduction

The present study uses theories of cognition to explore how time pressures can influence journalists as they make coverage decisions during a presidential campaign. Research on human cognition suggests there are two distinct cognitive modes in the human mind, typically referred to as System 1 and System 2 [1–3]. System 1 is a rapid, low-effort, and intuitive way of thinking, often based on emotions and habits. In contrast, System 2 processes information more slowly, analytically, and systematically to arrive at a conclusion. People spend most of their time engaged in System 1 thinking since it minimizes cognitive effort and alleviates time pressure through the use of heuristics. For example, people tend to overestimate the likelihood of events based on ease of recall of related examples [4]. When exposed to new information, people may not always engage in analytical thinking and often rely on the first piece of information even when it is irrelevant [5]. This tendency is not constrained to specific individuals but rather describes general patterns of human behavior. Although System 1 thinking can be prone to errors, people can operate naturally as cognitive misers and try to ‘satisfice’ perceived needs [6].

Insights from theories of thinking systems can help explain how journalists may not always use thorough, analytical reasoning and may engage in judgmental shortcuts when covering presidential campaigns. Journalists on the campaign trail often face time pressure as they need to distill unfolding events quickly during presidential primaries [7], party conventions [8], and presidential debates [9]. For many journalists under time pressure, one advantage of social media platforms such as Twitter lies in its fast dissemination of information [10, 11]. Twitter yields instant publication when events are still taking place, often serving “as the ‘first’ depository for news” ([12], p. 298) for many journalists who cover presidential campaign. While Twitter can help journalists share stories fast with a wider audience, it can accelerate time pressures on campaign reporters to cut corners and publish first. According to a study that interviewed journalists from various U.S. national, metropolitan, and local newspapers [13], Twitter had a direct impact on journalists’ workflow by promoting “speed-driven content through emerging news routines that demanded more frequent micro-updates” ([13], p. 231). In addition, Twitter’s cultural milieu of informality can lead journalists to take an informal, personalized, and less analytical approach. Studies have found that journalists often infuse humor and personal opinions in their tweets to engage with a wide audience and to establish personal branding [9, 14, 15]. Facing rapid streams of information coming through Twitter where more than 400 million tweets are generated per day [16], journalists feel compelled to adapt to the rhythms on Twitter and try to break news first in order to attract more views in a timely and personal manner.

This paper applies cognitive theories to examine when and how System 1 thinking can be evident in journalists’ minds through empirical investigations of their word choices across different media. Theories of the mind predict that journalists on the campaign trail may engage in System 1 thinking when they sift through volatile campaign events. Of particular interest to the current discussion is whether journalists are more likely to engage in System 1 thinking when they navigate Twitter than when they compose texts and scripts for traditional news media such as newspapers and broadcasts. While System 1 thinking can influence traditional journalistic outputs as reporting inevitably involves time pressure and uncertainty [17–19], it can be more amplified in the Twitter environment due to the platform’s emphasis on speed, brevity, and personal approaches [10–15, 20]. When time is pressing and deep thought is a luxury, System 1 thinking may dominate as reporters engage with Twitter.

To explore these research questions, we built a unique text corpus of journalistic outputs (*N* = 220,111) generated by a purposive sample of campaign reporters (*N* = 73) [21] over a 12-month period during the 2016 presidential election campaign from November 7 2015 to November 7 2016. Journalists’ writing and comments were produced in natural settings across various media such as papers (i.e., print or online newspapers and news magazines; 9,745,292 words across 17,272 articles), broadcasts (i.e., network television, cable, and radio; 237,583 words in 655 transcripts), and Twitter (i.e., 2,643,593 words from 202,184 tweets). Our text analyses suggest that journalists varied language by media in ways predicted by the System 1 and System 2 thinking [1–3]. This study is the first systematic attempt to explore how cognitive biases are manifested in journalists’ work across media through empirical investigations of the language of their reporting in large-scale text.

This study expands prior research in several ways. There has been growing recognition of the ways cognitive biases can be present in journalists’ work. Researchers note that journalism is inherently “institutionalized heuristic decision making” ([17] p. 193) since System 1 thinking is embedded in journalism practices due to the rapid nature of work and recurring deadlines [18]. Yet, cognitive influences on journalistic work have been relatively underexplored. A recent study [22] discussed the inevitable errors and cognitive biases in journalists’ work and suggested several ways professional journalists and educators could address the pervasive impact of cognitive biases on news reporting. While these studies highlight that cognitive biases can appear in the work of journalists, the impact of cognitive biases in journalistic work is often assumed or implied. This study empirically examines how System 1 thinking can be manifested in journalistic outputs.

This study also provides novel insights into the online information landscape by shifting attention from media users’ cognition to journalists’ cognition. Growing evidence suggests that media users rely on System 1 thinking when navigating social media platforms and facing a stream of updates and posts. To process information fast, readers often utilize heuristics such as image size or a ‘breaking’ label to identify important information on Facebook [23]. The architecture of social media where social boundaries are collapsed [24] can also promote System 1 thinking in readers’ minds, as evidenced by inattentiveness to source information [25]. Given the speed and context of the information rushing through on social media, there is less scope for elaboration in readers’ minds. What remains unclear is whether similar cognitive patterns operate among journalists. Do the time pressures of social media make System 1 thinking more evident in tweets than in texts and transcripts from traditional media? How might cognitive theories and heuristics help explain word choices and patterns in political coverage? Considering that time pressures on journalists can influence the quality of news reporting [26, 27], it is imperative to understand journalists’ cognitive mindsets and attitudes under time pressures.

Lastly, this study promotes a deeper understanding of journalists’ thinking through naturalistic assessments of large amounts of text. The majority of scholarly research on the cognitive process employed by journalists tends to be theoretical [17, 18], pedagogical [22], or evaluated through surveys [28], lab experiments [29], or a snapshot of a small number of mainstream news outlets [19, 30–33]. While these studies shed light on the role of cognitive biases in journalistic outputs, it remains unknown how journalists vary their word choices across media and how different use of the language can be related to journalists’ cognitive mindsets. This article examines journalists’ word choices and patterns in various forms of writing and comments generated in natural settings over extended time periods, opening a new frontier of research that explores the psychology of language through automated text analysis of journalistic outputs.

## Literature review

### Current perspectives on cognitive biases in journalism

While journalism research has traditionally emphasized social, cultural, and organizational contexts in the newsmaking process [34–36], there have been prior attempts to explore how individual journalists’ cognition can play an important role in their news decisions. One notable example comes from Stocking and Gross [18] who applied cognitive insights to journalists’ decision-making processes and explained how cognitive biases could influence their information gathering and news coverage practice. The researchers posited five cognitive tasks involved in the newsmaking process: categorization, theory generation, theory testing, information selection, and information integration. In categorization, journalists assess initial information, and during this process, various cognitive biases could influence journalistic decisions. For example, journalists might favor incoming information when it is related to recent memory [4]. After categorization, journalists select a certain angle (termed ‘theory generation’) and cover stories by choosing sources (referred to as ‘theory testing’). The theory generation and testing process can be affected by a variety of errors and cognitive biases, including confirmation bias where people seek and interpret information in ways that are consistent with their expectations and existing beliefs. For instance, journalists are likely to select information that supports their arguments and to discredit the source of disconfirming information. Lastly, journalists compose news stories by synthesizing all information they gathered, which might be affected by false causality or over-simplification of correlational events. This framework helps convey how “a variety of ways of thinking (indeed a variety of routine ways of thinking) that constrain one’s perceptions and interpretations of the world” ([18], p. 16) can affect journalistic decisions.

Building upon this framework [18], researchers examined how cognitive biases might be manifested in journalists’ use of language. Researchers [37] interviewed 11 reporters at a local newspaper in the Midwest and examined how journalists planned to cover stories they had not started formally reporting yet. Focusing on journalists’ words in interviews, the researchers examined if journalists brought assumptions to their stories (e.g., *theory*, *test*, *may*, *seems*, *whether*, *if*, *possibly*, *potentially*). Results suggest that only half of the story ideas (16 of 32) described by journalists contained alternative hypotheses, indicating confirmation bias embedded in journalists’ minds. Similarly, another study [19] investigated the impact of confirmation bias in news coverage, focusing on 3 local television news programs in Milwaukee, Wisconsin, about the federally mandated reformulated gasoline program enacted by the Clean Air Act in 1990. The researchers found that cognitive biases and errors, such as confirmation bias, shaped the ways reporters covered a health study about gasoline additives. Reporters’ selection of sources and interpretation of the health study were largely driven by their desire to support their theory that the health study was flawed. For example, journalists only referenced the sources that were critical of reformulated gasoline and omitted relevant information about the health study, such as rationales for random sampling and the discrepancy in health complaints between the control area and Milwaukee. A more recent study [22] about cognitive bias in journalism centered around a pedagogical approach and suggested several ways journalists and educators could address the inadvertent impacts of cognitive biases on news reporting. For instance, journalists are encouraged to think about counterarguments in their stories to surface potential confirmation bias. Recent developments in psychology to measure implicit biases about racial minority groups such as the Implicit Association Test (IAT) [38] can be also a useful way to enhance awareness among journalists about biases and cognitive errors that can inadvertently creep into their minds. These findings show that cognitive biases can shape how journalists select a certain angle, choose sources, and cover news stories.

Other scholars have tried to understand the cognitive process involved in journalists’ work in a broader sense. Using multiple surveys from journalists, one study [28] explored how psychological needs to preserve one’s existing beliefs played a pivotal role in journalistic decisions. Surveys suggest that more than the majority (51%) of German reporters indicated that they selected headlines, sources, and pictures that were congruent with their hypotheses and expectations. Another study [29] employed experiments to examine how journalists utilized various news sources when they wrote news stories. Journalists in the United States and China were asked to compose a news story in 30 minutes, using all the information provided. The stimulus information provided with the journalists contained 4 news elements associated with cognitive, cultural, and rational system levels, respectively. The findings suggest that despite the national and cultural differences, journalists spent more effort in processing cognitive news elements and included them more in the stories than other news elements. These studies indicate that journalists’ decisions in how they cover a story involve predictable cognitive processes, emphasizing the importance of understanding how journalists think when they write.

Another strand of research examines linguistic register variations in journalistic reports using Critical Discourse Analysis (CDA). Research in CDA views language as constituents and outcomes of economic, social, or political power inequalities [30, 31]. CDA has been increasingly embraced by media researchers who are interested in examining social contexts through critical investigations of linguistic patterns in journalistic discourse. For instance, one study [32] examined journalists’ use of ‘stance adverbs’ (e.g., *obviously*, *clearly*, *apparently*, *presumably*) in 18 sentences drawn from a variety of news stories produced by the United Press International wire service in 1991. The author found that stance adverbs were a textual strategy to support journalists’ arguments or to discount the legitimacy of claims made by other reporters. Another study [33] examined ‘empty signifiers’ (e.g., *change*, *hope*, *we*) in political reporting and their relation to populism. This work examined 62 news reports generated during the week after the delivery of the four separate speeches made by Barack Obama at the 2004 Democratic National Convention in Boston, the announcement speech in Springfield, Illinois, the race speech in Philadelphia, and the acceptance speech at the 2008 Democratic National Convention in Denver. Results suggest that populist discourse was manifested in the use of empty signifiers in news media coverage. In the context of reporting of international news, researchers [39] examined news discourses in 9 Belgian broadcast programs about natural disasters that occurred in Australia, the U.S., Indonesia, and Pakistan. Text analysis reveals that natural disasters in the U.S. and Australia were portrayed as important and worthy of attention. This contrasts with news coverage of the Indonesian and Pakistan disasters, which were described as involving distant ‘Others’ without cause for concern or actions. These findings show how the semantic or grammatical features of text can be a useful tool for understanding the social system and power hierarchy that journalists inhabit.

Research in various traditions have explored journalists’ cognitive processes and how cognitive biases can be manifested in their word choices. Building upon this prior research, the present study examines how cognitive biases can be embodied in journalistic outputs, focusing on potential differences across media and how the differences in journalists’ word choices can reflect distinct cognitive processes employed by journalists.

### System 1 thinking on Twitter

Emphasis in the Twitter community on immediacy can lead journalists to engage in System 1 thinking, which is characterized by low-effort, self-validating information processing driven by speed, emotions, and habits [1–3]. According to one study that examined real-time fact-checking activity among political journalists and commentators on Twitter during the 2012 U.S. presidential debates [40], the majority of political journalists’ Twitter activity did not involve fact-checking. While Twitter could be a useful avenue for quick fact-checking of candidate’s claims through hyperlinks to other sources of news and information, only 15 percent of journalists’ tweets included some form of fact-checking and presentation of evidence. Instead, journalists mainly engaged in posting opinionated tweets and commentary about the candidates’ claims.

In situations of crises and conflicts, System 1 thinking can be even more pronounced among journalists since they try to keep up with fast-changing discussions and revelations online. During the 2016 Brussels bombings, journalists tended to rely on user-generated content on Twitter in news items without engaging in basic verification procedures [41]. Another study [42] that examined media coverage of the Haiti earthquake in 2010 by the *Guardian*, BBC, and CNN, found similar results. The ‘tweet first, verify later’ approach was generally adopted by the news organizations, and only the BBC consistently sought to verify information on social media before it published news in its online version. These examples illustrate that when speed is crucial, it can pose a great challenge for journalists to adhere to deliberate, thorough, and systematic reasoning.

Interview evidence supports this notion of Twitter time pressures [12, 13]. When journalists described their decisions to deactivate or cut back on Twitter, they noted a change in their reporting. One reporter said that reducing Twitter use gave “his writing a different “accent” that’s more open to expressing ambivalence and exploring varied perspectives” ([20]). Another journalist indicated that deactivating Twitter helped them “draw from broader references and … think about things in a more contextual way than a reactive way” ([20]). In a study involving interviews with US newspaper journalists [43], reporters shared their concerns regarding the impact of the accelerated work pace brought by digital technology on the quality of reporting. One journalist commented “there will be more errors, we will do less editing, and we are willing to live with that” ([43] p. 77). Another echoed concerns about increasing time pressure on work: “they want us to do shorter, quicker pieces that really don’t have a lot of depth” ([43] p. 77). These remarks show that many journalists simply may not have the luxury of time to get context, diverse views, and background facts as they keep up with the rapid news cycles online. The quest for speed can be more pronounced on Twitter where the flow of information has tremendously accelerated due to the immediate nature of networked communication.

In addition to the accelerated tempo of Twitter, one of the notable characteristics of journalists’ use of Twitter is a shift towards the individualization of journalism. Studies suggest that journalists express themselves more spontaneously on Twitter to connect with the audience on a more personal level. Researchers [14] examining the most followed journalists as of 2009 found that many of the popular journalists on Twitter regularly used humor. Content analysis reveals that 22.5% of journalists’ tweets centered around humor. 20.2% of journalists’ tweets were focused on developing relationships with their audience and 15.9% were lifecasting, such as sharing personal anecdotes about their everyday life (e.g., where they had lunch). Journalists’ use of Twitter during the presidential election campaign has been also found to involve an informal and personal approach. According to one study [9] that examined how political journalists engaged with Twitter while covering the first 2012 presidential election debate between Mitt Romney and Barack Obama, approximately 20% of the journalists’ tweets contained jokes, indicating a departure from traditional news coverage of politics. Adapting to the Twitter community’s informal conversation styles, journalists take a more personal and casual rhetorical approach to build relationships with their audience.

Personalization of journalism on Twitter can be partly driven by self-promotion and personal branding efforts. Similar to the process observed in influencers and celebrities on Twitter [24], journalists have been found to utilize Twitter to promote their work and develop personal branding. Journalists often share links to their own news stories or work done by their co-workers [44], post backstage selfies with celebrities [45], and comment on praise and criticism they received from Twitter users [46]. This personalized discourse blurs professional and personal lines, creating a sense of intimacy and forming affiliations with followers. While journalists feel that there is a tension between individuals and organizations in establishing brand on Twitter [15], self-branding is now part of the everyday routine of many journalists and can lead to particular styles of writing focused on personal, informal, and less analytical approaches.

Inspired by these findings, this study posits that System 1 thinking can be more pronounced in journalists’ minds when they navigate Twitter than when they compose texts for traditional news media such as newspapers and news magazines. When journalists engage in System 1 thinking, we should see this reflected in their word choices. For instance, a study [47] examining clinician-patient consultation under different time settings (7 minutes versus 15 minutes) compared the primary linguistic features extracted from the transcripts of these interactions. The results demonstrate that when the clinicians were under time pressure, the resulting cognitive effects were evident in linguistic features such as using more direct language, exhibiting more emotion, and providing less evidence for their arguments. Similarly, we can expect when journalists write on Twitter, they are likely to engage in System 1 thinking and thus their language should reflect more emotion (**H1a**), certainty (**H1b**), and a focus on the present (**H1c**) than news articles.

**H1a-c:** Journalists will use words charged with more (a) emotion, (b) certainty, and (c) emphasis on the present on Twitter than when they write for papers (i.e., print or online newspapers and news magazines).

Furthermore, Twitter’s informal culture can lead journalists to rely on more personalized and casual language. A large body of research [48, 49] in the sociolinguistic tradition shows that situational characteristics and communicative purpose can lead to linguistic differences (termed ‘register variation’). For instance, the register of news is more abstract than that of fiction. This is reflected in the use of more conjuncts (e.g., *furthermore*, *therefore*) and more agentless passives (e.g., Michael *was awarded* a prize) in news than in fiction [48]. Recently, researchers examined linguistic variation in Donald Trump’s tweets posted between 2009 and 2018 [50]. Results suggest there are generally four dimensions of stylistic variation in Trump’s tweets related to conversational, campaigning, engaged, and advisory discourse. Trump’s use of Twitter to engage with his followers is reflected in frequent use of WH-questions (e.g., what, which) and private verbs (e.g., accept, assume, believe, check), which are considered rhetorical resources to acknowledge alternative positions and to invite feedback from his followers on the ongoing dialog. In this respect, it is expected that journalists will engage with more personal, informal, and casual language in tweets as they connect with their audiences on a more personal level [9, 14]. This can be manifested in frequent use of authentic language (**H1d**), fillers (e.g. *I mean*, *you know*; **H1e**), and Internet slang, commonly referred to as netspeak (e.g., *thx*, *btw*, *lol*; **H1f**). In contrast, journalists are expected to engage less with informal language when they write for newspapers and news magazines because these traditional media tend to take a relatively formal rhetorical approach and involve multiple stakeholders such as editors and public relations managers, leading to abstract language [48].

**H1d-f:** Journalists will use more (d) authentic language, (e) fillers and (f) netspeak on Twitter than when they write for papers (i.e., print or online newspapers and news magazines).

In contrast, System 2 thinking is less likely to be prevalent in journalists’ minds when they navigate Twitter due to its emphasis on speed, brevity, and informal conversational styles. Hence, journalists are less likely to use analytical words (**H1g**) or provide numerical evidence (**H1h**) when they tweet than when they compose stories for papers.

**H1g-h**: Journalists will use fewer (g) analytic words and (h) numerical terms on Twitter than when they write for papers (i.e., print or online newspapers and news magazines).

Next, we turn to broadcasts, another form of traditional news media, such as network television, cable, and radio. We hypothesize that System 1 thinking is more pronounced in journalists’ minds when they tweet than when they compose scripts for informational broadcast programs that tend to have relatively more formalized information production process such as programming schedules and editorial decisions. Similar to the investigation of linguistic differences between Twitter and papers, differences in journalists’ word choices on Twitter and broadcasts are examined through the following hypotheses:

**H2a-c:** Journalists will use words charged with more (a) emotion, (b) certainty, and (c) emphasis on the present on Twitter than when they appear in broadcasts (i.e., network television, cable, and radio).
**H2d-f:** Journalists will use more (d) authentic language, (e) fillers and (f) netspeak on Twitter than when they appear in broadcasts (i.e., network television, cable, and radio).
**H2g-h:** Journalists will use fewer (g) analytic words and (h) numerical terms on Twitter than when they appear in broadcasts (i.e., network television, cable, and radio).

### Anchoring in campaign coverage

While System 1 thinking may be more pronounced on Twitter, snap decisions are often involved in daily journalism practice [13, 17–19]. Hence, we can expect that journalists on the campaign trail will use mental shortcuts associated with System 1 thinking in their coverage across all media. Studying how journalists process information under uncertainty and time constraints, we focus on one type of heuristic, *anchoring*, that has been proven especially robust in explaining decisions in many different domains of choice.

Anchoring refers to a phenomenon where people’s estimates of uncertain quantities are biased by the consideration of prior information. In a classic study by Tversky and Kahneman [5], participants were asked to provide an estimation of the percentage of the United Nations members that were African countries with reference to numbers between 0 and 100 randomly generated by spinning a wheel. Participants were then asked to consider whether their guess was higher or lower than the reference value presented before they formulated an answer. Although the starting numbers were given arbitrarily, the initial numbers had a marked effect on participants’ estimates. The median estimates of those who received 10 and 65 as starting points, respectively, were 25% and 45%. Results showed that judgments under uncertainty may be guided by salient numbers even if these are determined at random.

When estimating uncertain situations, people often anchor on information that comes to mind and gradually adjust their estimate until a plausible estimate is reached. Anchoring occurs if the estimate is drawn towards the anchor and the adjustments end prematurely. For example, when participants were asked to estimate the average length of the Mississippi River, they gave a larger estimate when they were asked if it was longer or shorter than 2,000 miles than when they were asked to guess in comparison to 70 miles [51]. In addition to insufficient adjustment, another mechanism of anchoring occurs as a priming effect. One experimental investigation [52] found that when participants were shown subliminal anchors while they were thinking about the average price of a car, their estimates were assimilated to a high or low anchor that was presented outside of awareness. Recent research [53] has also shown that anchors can come in many, not necessarily numerical, forms.

Altogether, findings on anchoring suggest that one of the most striking characteristics of human judgment lies in its comparative nature. Individuals rely on comparisons to construct their evaluations. Building upon these findings, we propose that when journalists covered the 2016 U.S. presidential election, they anchored on a pertinent context, particularly their experience of presidential election coverage prior to 2016. Journalists’ experience in covering previous presidential elections became a judgmental anchor when they tried to describe the unfolding 2016 presidential campaign. Among a wide number of variables that could factor into a journalist’s news decision, covering a major election can be the assignments of a lifetime for many journalists and may provide a reference point in their later reporting [36]. Differing life and career experiences provide journalists with different anchors, which means their stories will differ in language and approach. While all reporters in the 2016 campaign had the opportunity to draw upon historical references in their coverage, those who lived this history by covering previous campaigns were more likely to anchor on the past to cover the present.

**H3:** Journalists who had covered presidential elections prior to 2016 are more likely to invoke previous political events in their news reports about the 2016 race than those without any experience of campaign coverage.

This study also posits that contrary to prior political events that only some journalists had covered, anchoring to present political events should not be different depending on journalists’ experience. For example, the 2016 primaries can serve as a reference point because every 2016 campaign reporter presumably followed the unfolding 2016 primaries then. Therefore, we can expect that journalists would invoke the 2016 primaries across the course of the year to similar degrees regardless of their campaign experiences prior to 2016. A comparison between past and present political events allows us to test if anchoring on the past was likely to come from journalists’ experience, not from their individual preferences about how to cover politics and elections.

**H4:** Regardless of their experience in covering presidential elections prior to 2016, journalists will make comparable references to the 2016 primaries in their news reports about the 2016 race.

## Methods

### Ethics statement

The Institutional Review Board of Stanford University determined that this study posed no more than minimal risk and granted approval (assurance number: FWA00000935).

### Data overview

#### Sample

We identified a purposive sample of campaign reporters, drawing from a Politico survey [21] administered in March 2016 to 82 campaign reporters who were covering the presidential election then. Politico reported results describing reporters’ political party registration (21% Democratic, 8% Republican, 22% Independent, 49% not registered), gender (61% male, 39% female), experience (63% covered at least one prior presidential election, 37% no prior experience), and platform (i.e., print, web, magazine, television, and radio). We adopted this sample of journalists for analysis. Note that it is not representative of all professional journalists. It does though provide a snapshot of reporters covering the 2016 presidential election, and there is variance among these 82 reporters in terms of campaign coverage experience, platform, party affiliation, gender, and age.

#### Text corpus

In the following analysis, we mainly used two sets of text generated through coverage in traditional media and on Twitter from the 82 campaign reporters in the Politico survey [21]. The news corpus was generated as follows. Out of the 82 reporters, 64 were from papers and 62 journalists’ articles were available from either news outlets or LexisNexis at the time of data collection. We collected all the available news articles generated during the 2016 presidential campaign season from November 7 2015 to November 7 2016. This procedure yielded a total of 9,745,292 words in 17,272 articles written by 62 journalists for print or online newspapers and news magazines.

Next, we collected the transcripts of the broadcast informational programs where the sample journalists appeared, often as commentators or guests, during the 12-month sampling period. Following the same procedure taken by Hamilton [54], we downloaded transcripts from the LexisNexis database and then edited the transcripts so we could examine only the target journalists’ spoken words and remove the words of other commentators and guests. We collected a total of 655 transcripts that contained 237,583 spoken words of 33 journalists from network television, cable, and radio.

We scraped all tweets generated by the sample journalists during the campaign season, using Twitter API. In our sample data, 59 reporters posted at least one tweet during the 12-month sampling period and still had tweets visible at the time of data collection. If a tweet was written in a language other than English or simply retweeted someone else’s tweet without any additional comments from the sample journalists, we did not include it in the analysis. As a result, a total of 2,643,593 words in 202,184 tweets posted from the 59 journalists’ Twitter accounts were included in the analysis.

Lastly, we collected additional information about the sample journalists. We used online sources, including news outlets’ biographies, individual journalists’ portfolio websites, LinkedIn, and Muck Rack. Through these sources, we gathered information about journalists’ background such as gender, estimated age at the time of 2016, and Twitter accounts. At the end of this procedure, we excluded 9 journalists from our sample because neither their news articles nor tweets were available for the analysis. A total of the 73 journalists ultimately constituted our sample of reporters whose articles from media (papers and/or broadcasts: *n* = 71) and/or tweets (*n* = 59) were available for data collection.

### Data cleaning and measures

#### Data cleaning

With two sets of text collected from news outlets and Twitter, we conducted basic text processing such as removing HTML special entities. For the Twitter corpus, we removed retweeted messages, mentions of username, and any external website links so we could investigate only the words of the sample journalists. The final text corpus was composed of a total of 12,626,468 words from papers (*n* = 9,745,292 words), broadcast transcripts (*n* = 237,583 words), and Twitter (*n* = 2,643,593 words). While there is variance in the number of words across media, we obtained large amounts of text from each of the three media, allowing us to extract robust results from each type of media and examine differences in word choices across media.

#### System 1 thinking across media

To explore how System 1 thinking was reflected in language use across different media, we used a standard text analysis tool called Linguistic Inquiry and Word Count (LIWC) [55]. The LIWC program provides a variety of psychologically meaningful word dictionaries across more than 90 different language dimensions (e.g., certainty: ‘always, never’, positive emotion: ‘love, nice, sweet’). It processes each word in an input file and searches its dictionary file for a match. At the end of this procedure, it calculates a relative frequency of the word categories to total number of words in each text input file. LIWC has been widely utilized in various research that examines cognitive and emotional dimensions in a wide variety of forms of media, such as blogs, novels, natural speech, newspapers such as the *New York Times*, and Twitter [56–58].

Specific to our examination of how journalists varied language across media, we focused on the categories that are likely to be indicative of a journalist’s cognitive and emotional orientation. Table 1 shows the LIWC indicators we analyzed and their examples in relation to the hypotheses these variables are associated with. In testing the hypotheses, we compared the mean LIWC word scores with a series of two sample *t-*tests. To limit the potential for false-positive results stemming from multiple comparisons, we set a conservative limit for reporting of results based on Bonferroni correction [59] (critical *α* = .006).

**Table 1:** Word categories for System 1 thinking on Twitter.

LIWC categories	Examples^a^	Papers (newspaper, magazine) vs. Twitter	Broadcasts (network, cable, radio) vs. Twitter
Positive emotion	love, nice, sweet	H1a: Papers < Twitter	H2a: Broadcasts < Twitter
Negative emotion	hurt, ugly, nasty	H1a: Papers < Twitter	H2a: Broadcasts < Twitter
Certainty	always, never	H1b: Papers < Twitter	H2b: Broadcasts < Twitter
Present focus	today, is, now	H1c: Papers < Twitter	H2c: Broadcasts < Twitter
Authentic	Summary metrics^b^	H1d: Papers < Twitter	H2d: Broadcasts < Twitter
Fillers	Imean, youknow	H1e: Papers < Twitter	H2e: Broadcasts < Twitter
Netspeak	btw, lol, thx	H1f: Papers < Twitter	H2f: Broadcasts < Twitter
Analytic	Summary metrics^b^	H1g: Papers > Twitter	H2g: Broadcasts > Twitter
Quantifier	few, many, much	H1h: Papers > Twitter	H2h: Broadcasts > Twitter

^a^Examples of word lists were drawn from Pennebaker et al. [60].

^b^According to the developers of the LIWC software [61], summary metrics such as authenticity [62] and analytical thinking [58] are derived from previously published findings and converted to percentiles based on standardized scores from large comparison samples.

#### Anchoring to political events

We compiled a list of words relating to previous political events (**H3**) and current political events (**H4**). As shown in Table 2, the lists of words relating to previous political events include candidates who ran for the 2012 presidential election (i.e., Obama, Biden, Romney, Ryan) and prior five presidential elections (e.g., Obama, Romney, McCain, George Bush, Kerry, Gore, Bill Clinton). For comparison purposes, word lists for the 2016 primaries were also generated, including a general vocabulary for primary elections (i.e., primary, caucus, nominee; adapted from Conway et al. [7]) and candidates (e.g., Hillary Clinton, Sanders, Trump, Cruz, Rubio). Note that the vast majority (90.5%) of the sample journalists who had experience in election coverage had covered five or less than five presidential elections prior to 2016.

**Table 2:** Word lists for anchoring hypotheses.

Hypotheses	Political Events	Words
Journalists who had covered presidential elections prior to 2016 are more likely to invoke previous political events in their news reports about the 2016 race than those without any experience of campaign coverage (**H3**).	2012 presidential election	Obama^a^, Biden, Romney^a^, Ryan
	Past five presidential elections (1992, 1996, 2000, 2004, 2008)	Obama^a^, Romney^a^, McCain, George Bush, Kerry, Gore, Bill Clinton, Dole, Perot
Regardless of the experience of presidential election coverage prior to 2016, journalists will make comparable references to the 2016 primaries in their news reports about the 2016 race (**H4**).	2016 primaries	primary, caucus, nominee, Hillary Clinton, Sanders, O’Malley, Trump, Cruz, Rubio, Kasich, Carson, Jeb Bush, Paul, Huckabee, Fiorina, Christie, Gilmore, Santorum

^a^Barack Obama and Mitt Romney were included as keywords for both the 2012 presidential election and the past five presidential elections because they ran in the 2008 and 2012 elections.

To see what anchoring means in practice, consider news articles written by two journalists in our sample about appeals to Hispanic voters. One demonstrates anchoring to the 2012 presidential election while the other has no anchoring:

Anchoring: “That announcement follows skittish Democrats chastising the campaign in a

*Washington Post* report for starting a concerted advertising campaign later than President Obama did in his 2012 race.” (*BuzzFeed News*, September 22 2016)

No anchoring: “After insisting for more than a year that all illegal immigrants “have to go,” Trump met with a newly created panel of Hispanic advisers on Saturday and asked for other ideas—making clear that his position is not finalized, according to two attendees.” (*The Washington Post*, August 21 2016)

The central focus of the analysis of anchoring bias was to examine if there were differences in the number of references to previous political events, depending on journalists’ prior experience of covering presidential elections before 2016. The anchoring bias hypothesis (**H3**) posits that journalists who had covered presidential elections prior to 2016 are more likely to make references to words related to the 2012 presidential election or the past five presidential elections in their news reports about the 2016 race, compared to journalists without any experience of campaign coverage before the 2016 election. In contrast, references to the 2016 primaries should not vary regardless of journalists’ prior experience of campaign coverage because all campaign journalists in the study sample presumably followed the 2016 primaries that were taking place then (**H4**). To control for the length of news reports, we computed average references to each of these political events (i.e., 2012 presidential election words, past five presidential elections words, and 2016 primaries) per 1,000 words. We then conducted a series of two sample *t*-tests to examine differences in the average number of references to these political events according to journalists’ campaign coverage experiences. To counteract the problem of multiple comparisons, we adopted a conservative approach and applied Bonferroni correction [59] (critical *α* = .017).

## Results

### System 1 thinking on Twitter

We hypothesize that when journalists are under the time pressures of Twitter, they will engage in System 1 thinking characterized by an emotional and self-validating information process that focuses on the present moment rather than a longer time horizon. Twitter’s informal discourse can lead journalists to adopt more casual conversational styles in their tweets. Furthermore, journalists on Twitter are less likely to rely on System 2 thinking that processes information analytically and numerically than they when they generate content for traditional media such as papers and broadcasts. In order to explore these hypotheses, we examined if there was any meaningful distinction in terms of emotional and cognitive words used by journalists when they navigated different media. A Bonferroni correction [59] was applied for a series of two sample *t*-tests before analyzing the results for significance (critical *α* = .006). Full results are reported in Table 3.

**Table 3:** Differences in language use across media.

LIWC Categories	Papers (newspaper, magazine)	Broadcasts (network, cable, radio)	Twitter	Difference of Means					
				Papers—Twitter			Broadcasts—Twitter		
	M (SD)	M (SD)	M (SD)	*Mean diff.*	*t*	*d*	*Mean diff.*	*t*	*d*
**Summary Language Variables**									
Analytical thinking	89.56 (11.50)	61.00 (22.20)	71.58 (32.97)	17.98	157.44**	0.73	-10.58	-12.16**	0.38
Authenticity	13.57 (12.58)	17.17 (16.44)	31.11 (36.29)	-17.54	-140.12**	0.65	-13.94	-21.53**	0.49
**Linguistic Dimensions**									
Positive emotion	1.96 (1.18)	2.35 (2.01)	3.39 (9.03)	-1.43	-65.23**	0.22	-1.04	-12.90**	0.16
Negative emotion	1.34 (1.09)	1.23 (1.13)	1.72 (5.49)	-0.38	-25.77**	0.10	-0.49	-10.75**	0.12
Certainty	0.53 (0.49)	0.96 (0.97)	0.87 (4.11)	-0.34	-35.07**	0.12	0.09	2.33	0.03
Present focus	7.47 (2.62)	12.90 (4.65)	10.04 (10.15)	-2.64	-85.34**	0.35	2.86	15.65**	0.36
Fillers	0.00 (0.03)	0.00 (0.03)	0.04 (1.52)	-0.04	-12.15**	0.04	-0.04	-11.70**	0.04
Netspeak	0.10 (0.27)	0.05 (0.15)	0.97 (7.00)	-0.87	-55.85**	0.18	-0.92	-55.82**	0.19
Quantifier	1.44 (1.17)	2.05 (1.44)	1.26 (3.65)	0.18	15.13**	0.07	0.79	13.94**	0.29
Number of reports	17,272	655	202,184						
Number of words	9,745,292	237,583	2,643,593						

*p < .006 (Bonferroni critical *α* = .006)

**p < .001

Results provide evidence of System 1 thinking in journalists’ tweets, compared to news articles. For instance, journalists infused more emotions in their tweets, both positive emotion, *t*(198,298) = -65.23, *p* < .001, Cohen’s *d* = 0.22, and negative emotion, *t*(123,604) = -25.77, *p* < .001, Cohen’s *d* = 0.10, when they tweeted than when they crafted news stories for papers (**H1a**). The sample journalists also showed more certainty in their opinions (**H1b**), *t*(207,227) = -35.07, *p* < .001, Cohen’s *d* = 0.12, and focused more on the present (**H1c**), *t*(79,067) = -85,34, *p* < .001, Cohen’s *d* = 0.35 in tweets than in news articles they composed. While effect size was generally small, these findings consistently support **H1a-c**, which posit that journalists’ tweets will contain more words that evoke emotions, indicate writers’ certainty, and are focused on the present than the news articles they write for papers.

Next, we investigated whether Twitter’s informal conversational styles were manifested in journalists’ word choices such as language that indicates authenticity (**H1d**), fillers (**H1e**), and netspeak (**H1f**). Results offer consistently supporting evidence for this projection. Compared to the news articles they wrote for papers, journalists’ tweets contained more words that indicated authenticity *t*(48,458) = -140.12, *p* < .001, Cohen’s *d* = 0.65. Use of fillers, *t*(203,662) = -12.15, *p* < .001, Cohen’s *d* = 0.04, and netspeak, *t*(208,740) = -55.85, *p* < .001, Cohen’s *d* = 0.18, were also more prevalent in tweets than in stories they composed for newspapers and news magazines. These findings suggest that Twitter’s casual conversational styles led journalists to take a more personal and informal rhetorical approach (supporting **H1d-f**). It is worth noting that the differences in linguistic patterns were especially pronounced in authenticity as indicated by the relatively large effect size, suggesting that journalists connected with their audiences on a more personal level on this social media platform.

Results also suggest that journalists were less likely to engage in System 2 when they were on Twitter (see Table 3). We hypothesized that System 2 thinking is less likely to be employed by journalists when they were on Twitter due to Twitter’s emphasis on speed, spontaneity, and informal cultural milieu. Since System 2 thinking is related to systematic processing of information based on reasoning and evidence, it was posited that use of words that denote analytical thinking (**H1g**) and numerical evidence (**H1h**) would be less common in tweets than newspaper articles. Consistent with these hypotheses, journalists used significantly fewer words that indicated analytical thinking, *t*(47,982) = 157.44, *p* < .001, Cohen’s *d* = 0.73, and fewer quantifying terms, *t*(54,964) = 15.13, *p* < .001, Cohen’s *d* = 0.07, in tweets than in their news articles. Effect size suggests that linguistic patterns of analytical thinking were especially distinct between Twitter and papers. Overall, these results suggest that the language in journalists’ tweets was largely related to System 1 thinking, relative to the words they used for newspapers and news magazines (supporting **H1g-h**).

The differences between Twitter and broadcasts are generally in the same direction as those between Twitter and papers. As can be seen in Table 3, journalists used more emotional words on Twitter than when they appeared in broadcast programs (**H2a**)–either positive emotion, *t*(742.27) = -12.90, *p* < .001, Cohen’s *d* = 0.16, or negative emotion, *t*(757.19) = -10.75, *p* < .001, Cohen’s *d* = 0.12. Also, journalists made fewer references to numerical terms in tweets than they spoke for broadcasts (**H2h**), *t*(681.66) = 13.94, *p* < .001, Cohen’s *d* = 0.29, indicating System 1 was likely activated on Twitter. While effect size was generally small, these findings provide partial support for System 1 thinking in journalists’ minds when they navigated Twitter than when they spoke for broadcasts (supporting **H2a** and **H2h**).

Consistent with the hypotheses, journalists used more informal language on Twitter than when they appeared in broadcast programs. Journalists’ tweets included more authentic language (**H2d**), *t*(674.81) = -21.53, *p* < .001, Cohen’s *d* = 0.49, fillers (**H2e**), *t*(46,511) = -11.70, *p* < .001, Cohen’s *d* = 0.04, and netspeak (**H2f**), *t*(37,146) = -55.82, *p* < .001, Cohen’s *d* = 0.19, than their spoken words in broadcast programs. Similar to the linguistic differences between Twitter and papers, the levels of authenticity in language were clearly divided between Twitter and broadcasts as indicated in relatively large effect size. The results suggest that journalists used informal language as they adapt to Twitter’s networked and personalized environment (supporting **H2d-f**).

Noticeable differences also emerged (see Table 3). In contrast to the patterns we observed between papers and Twitter, there was no significant difference in certainty between broadcasts and Twitter (**H2b**), *t*(732.35) = 2.33, *p* = .02 (Bonferroni correction: critical *α* = .006). Journalists also appeared to focus less on the present (**H2c**), *t*(647.37) = 15.65, *p* < .001, Cohen’s *d* = 0.36, and engaged in more analytical thinking (**H2g**), *t*(663.37) = -12.16, *p* < .001, Cohen’s *d* = 0.38, when they tweeted than when they spoke for broadcasts. These results indicate that the psychology of language is not as clearly divided on broadcasts and Twitter as it is between papers and Twitter (rejecting **H2b-c** and **H2g**).

To probe deeper into different word usage patterns in line with System 1 and System 2 thinking [1–3], we examined which words were used similarly (or differently) across media. This exploratory analysis focused on a subset of journalists (*n* = 17) who generated outputs across all the three media of our interest (i.e., papers, broadcasts, and Twitter). By investigating word choices of the journalists who navigated the three media, this supplemental probing helps understand how journalists engaged with System 1 thinking as reflected in the language. The descriptive statistics of the outputs of the selected journalists are shown in Table 4.

**Table 4:** Number of observations of the selected journalists (*n* = 17).

	Papers (newspaper, magazine)	Broadcasts (network, cable, radio)	Twitter
**Number of reports**	3,128	238	81,844
**Number of words**	1,636,531	44,245	768,054

**Fig 1:**
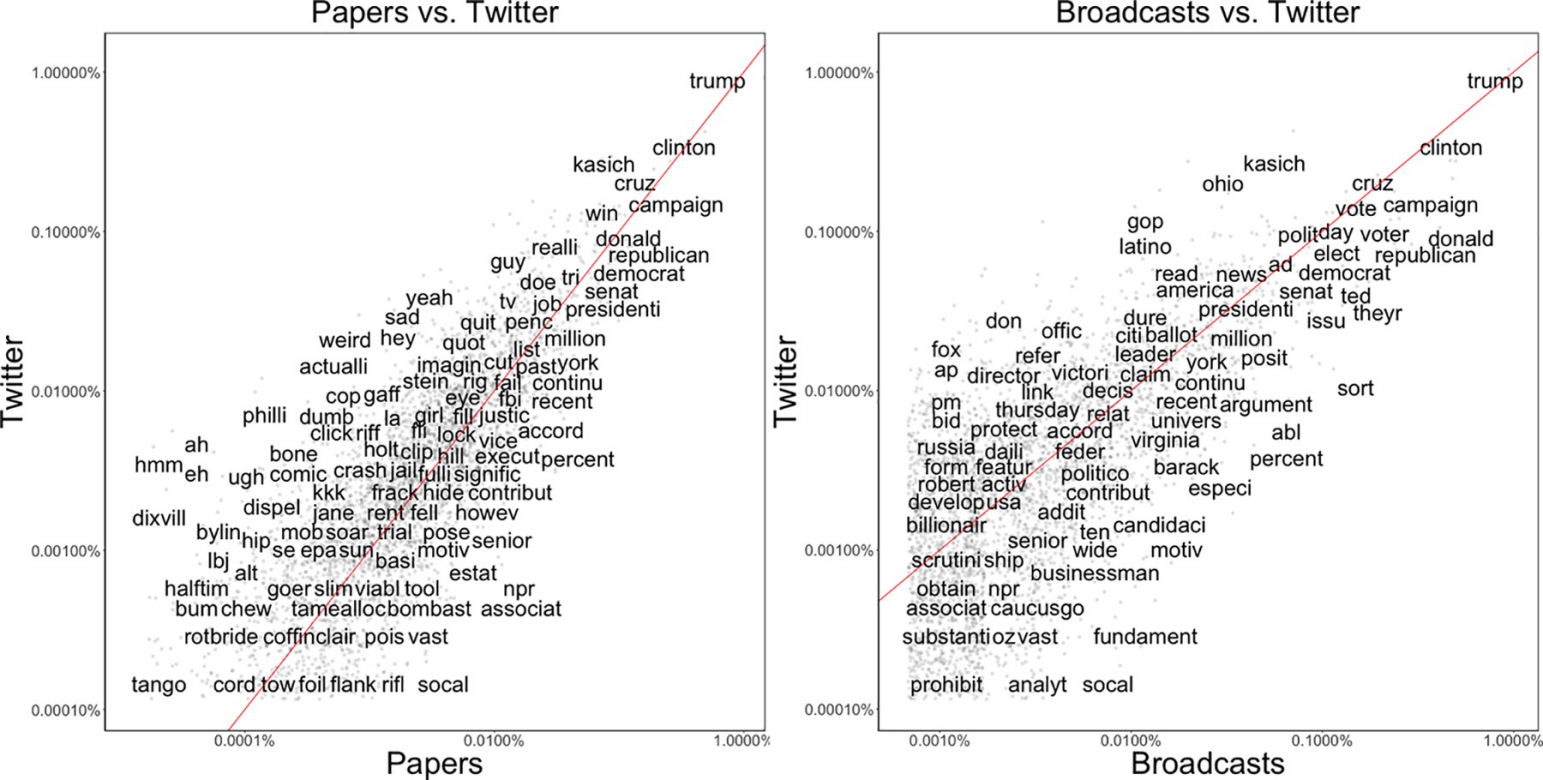
Word frequency by media.

Fig 1 represents word frequency averaged for each journalist across different media. Words were processed by stemming in which inflections and derivationally related forms of each word were reduced into its common base forms (e.g., languages -> languag, programming -> program, studies -> studi). Word frequency was normalized to the total number of words used by each journalist. For the sake of simplicity, the top 3,000 words that appeared frequently in the samples were indicated in the figure and words at the low end of frequency were blurred. Words near the red line were used with approximately equal frequency by two media in comparison–papers vs. Twitter (the left panel of Fig 1) and broadcasts vs. Twitter (the right panel of Fig 1). Words far away from the line are used much more by one medium compared to the other.

Fig 1 demonstrates that there are notable differences as well as similarities in journalists’ word choices across media in ways predicted by System 1 and System 2 thinking [1–3]. Candidate names (e.g., Donald, Trump, Clinton, Cruz, Sanders) and election-related words (e.g., campaign, presidential, vote, politics) appeared frequently across all media. As shown in the left panel of Fig 1, journalists infused their tweets with emotions (e.g., sad, weird, dumb) and used a casual, personal tone (e.g., ah, hmm, yeah, hey), compared to their writing for papers. Journalists appeared to take a more analytical approach when they composed texts for papers as indicated by referring to numerical evidence (e.g., percent) and to sources (e.g., according). Similar patterns emerged in journalists’ spoken words in broadcast programs versus Twitter (see the right panel of Fig 1). Journalists relied on numbers (e.g., percent, ten, million) and used words that could suggest analytical thinking (e.g., issue, motive, analytic) when they spoke for broadcast programs. Compared to their spoken words, journalists on Twitter seemed to focus on unfolding campaign events (e.g., GOP, Ohio, Russia) in a networked and immediate manner. For instance, they referred to other news sources (e.g., Fox, AP) and provided links to the news (e.g., link, read) in their tweets. This can be in part driven by journalists’ efforts to engage with their audience or to promote their own works or news stories covered by their co-workers [14, 44–46]. Overall, these patterns suggest that journalists engaged in System 1 thinking when they navigated Twitter than when they composed texts and transcripts for traditional news media such as newspapers and informational broadcast programs.

### Anchoring in campaign coverage

In addition to the differences in journalists’ language between media, we explored how System 1 thinking might be generally manifested across all media through an investigation of the anchoring hypotheses (**H3** and **H4**). We counted the number of references to the words relating to political events (see Table 2 for the word lists) and then normalized it to every 1,000 words to control for different lengths of news articles. The average references were compared between the journalists who had covered at least one previous presidential election before 2016 and those who had not. A Bonferroni correction [59] was applied for a series of two sample *t*-tests for multiple-comparison correction (critical *α* = .017).

**Fig 2:**
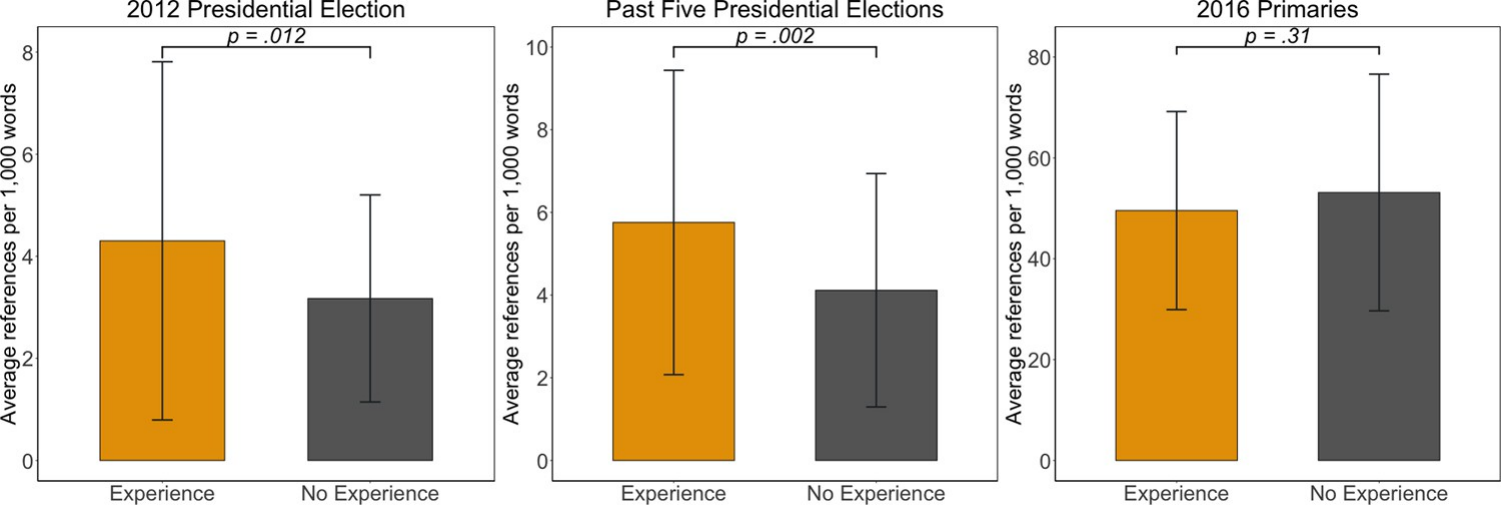
Anchoring on prior political elections. Two sample t-tests for difference between means. Bonferroni critical α = .017; *p < .017, **p < .01.

As shown in Fig 2, results suggest strong evidence of anchoring bias. In every 1,000 words of news reports and tweets, journalists who had covered previous presidential campaigns made significantly more references to the 2012 presidential elections (*M* = 4.30, *SD* = 3.51) than those without any prior experiences (*M* = 3.17, *SD* = 2.03), *t*(151.73) = -2.53, *p* = .012, Cohen’s *d* = 0.39 (see the left panel of Fig 2). As demonstrated in the center panel of Fig 2, similar patterns held for the past five presidential elections (*t*(136.88) = -3.09, *p* = .002, Cohen’s *d* = 0.50). Compared to journalists who had not covered any prior presidential elections (*M* = 4.12, *SD* = 2.82), journalists with experiences of campaign coverage used more words relating to the past five presidential elections in their news reporting of the 2016 race (*M* = 5.76, *SD* = 3.68). These results with moderate effect size suggest that anchoring on the past occurred in news coverage of the 2016 race according to journalists’ prior experience of presidential campaign coverage (supporting **H3**).

Reflecting interest in the currently evolving political events, the average references to the 2016 primaries were markedly higher (prior experience: *M* = 49.56, *SD* = 19.65, no experience: *M* = 53.14, *SD* = 23.47) than those to other previous political events (see the right panel of Fig 2). Consistent with the anchoring hypothesis, there was no significant difference in invoking the 2016 primaries depending on reporters’ campaign experiences (supporting **H4**), *t*(152) = 1.01, *p* = .31. Unlike the 2012 or past five presidential elections which only some of our sample journalists had covered, the 2016 primaries unfolded while all sample journalists were following the campaign. It is understandable that all reporters in our sample covered the 2016 primaries and used comparable numbers of words relating to the primaries in reporting of the 2016 race. These findings indicate that the differences in anchoring on the 2012 presidential election and the past five presidential elections were likely associated with journalists’ prior experience, not with their reporting styles in covering politics.

Supplementary probing was conducted to investigate further the use of the anchoring heuristic and control for potential compounding factors such as journalists’ age, gender, and type of media they were primarily associated with. Considering the distribution of data, we constructed two negative binomial regression models [63]. Full results are reported in Table 5. Note that observations were restricted to the news reports generated by the sample journalists through their primary news outlets in this person-level supplemental analysis. Tweets and news reports generated through non-primary outlets, mostly as guests or commentators, were dropped from the analysis.

**Table 5:** Impact of a journalist’s experience of presidential campaign coverage on reporting the 2016 presidential election.

	References to 2012 presidential election			References to past five residential elections		
	B (SE)	*e* ^*B*^	*95%CI for e* ^*B*^	B (SE)	*e* ^*B*^	*95%CI for e* ^*B*^
**Journalists’ prior experience** ^**a**^	0.39** (0.14)	1.47	[1.11, 1.94]	0.36** (0.12)	1.43	[1.13, 1.79]
**Papers** ^**b**^	0.62** (0.23)	1.85	[1.16, 2.86]	0.54** (0.19)	1.72	[1.18, 2.45]
**Age**	-0.01 (0.01)	1.00	[0.98, 1.01]	0.00 (0.01)	1.00	[0.99, 1.01]
**Female**	-0.12 (0.13)	0.89	[0.69, 1.15]	-0.09 (0.11)	0.92	[0.74, 1.13]
**Intercept**	-13.03*** (0.37)	0.00	[0.00, 0.00]	-12.91*** (0.30)	0.00	[0.00, 0.00]
**Number of observations**		71			71	
**Log likelihood**		-406.36			-415.04	
**AIC**		822.71			840.09	

*Note*. B: Negative binomial regression coefficients; SE: Standard errors; e^B^: Exponentiated coefficients; AIC: Akaike Information Criterion; *p < .05

**p < .01

***p < .001

^a^Reference category: no experience covering presidential campaigns before 2016.

^b^Reference category: broadcasts (i.e., network television, cable, and radio).

Results reinforce the points that journalists anchor on the past in reporting of the 2016 race (see Table 5). When controlling for age, gender, and type of media, journalists who had covered presidential elections before 2016 made more references to the 2012 presidential election (B = .39, *p* < .01) and the past five presidential elections (B = .36, *p* < .01), compared to those who had not covered prior presidential campaigns. In other words, while holding the other variables constant in the model, journalists with experience in campaign coverage before 2016 are expected to make references to the 2012 presidential election when they write about the 2016 race 1.47 times more in every 1,000 words than journalists without prior experience. Similarly, when the other variables are held constant in the model, journalists who had covered presidential election prior to 2016 are expected to make references to the past five presidential elections 1.43 times more in every 1,000 words of news reports they generated, compared to those without campaign coverage experience. Thus, we find consistently strong evidence for anchoring on prior experience in reporting news.

## Discussion

Theories of the mind and memory predict how people react to time pressure and uncertainty, and in this paper, we use ideas about cognition to explain reporters’ political coverage. Leveraging a large-scale real world setting dataset of text corpus (*N* = 220,111) that contains news articles, broadcast transcripts, and tweets generated over extended time periods during the 2016 U.S. presidential election campaigns, we examined when and how System 1 thinking [1–3] can be manifested in journalistic outputs. Results show that relative to their work in papers, reporters were significantly more likely to use language that was emotional and focused on the present when they were on Twitter. Journalists’ language in tweets also contained more certainty but included fewer analytical words and fewer numerical terms than their news articles, suggesting self-validating and intuitive reasoning. There were some linguistic differences in terms of conversational styles between Twitter and papers. Journalists used more authentic language and informal words such as fillers (e.g., *I mean*, *you know*) and netspeak (e.g., *thx*, *btw*, *lol*) when they tweeted, than they wrote for papers. Albeit less stark, differences in language use between Twitter and broadcasts were generally similar to those between Twitter and papers. Notable similarities emerged across media as well. We found that campaign journalists generally relied on their prior experience [5, 51–53] in election coverage as they tried to describe and explain unfolding events. Overall, these findings indicate that journalists routinely engage in System 1 thinking in covering the evolving world of presidential campaigns, and System 1 thinking can be especially amplified in journalists’ minds when they navigate Twitter to engage with their audiences in a fast and personalized manner.

### Contributions

Building upon insights from psychology, social scientists have documented how cognitive biases play a significant role in political decision making. Empirical studies have demonstrated how motivated reasoning is evoked when voters make political judgments [64], when politicians face information contradictory to prior attitudes [65], and when regulators make policy decisions [66]. While research on how cognitive biases affect voters, politicians, and policymakers is robust, news media have been considered only as a source of the message that people process in biased manners, and empirical work examining the role of cognitive biases in journalism is sparse. The current study sheds new light on how cognitive biases in journalists can shape the way they cover a story.

There is a growing body of work on the impact of accelerating time pressures on journalists [10–13]. Researchers have found that reporters under increasing time pressure relied on fewer sources, conducted less cross-checking, and were dependent on public relations and politicians for news sources [26, 27]. Our results show that though System 1 thinking is inherently embedded in journalism practices [17–19], the emphasis on speed in social media can lead System 1 thinking to be more pronounced in journalists’ minds. These findings contribute to a more nuanced understanding of how journalists as human decision makers react to events and how the current media landscape might further lead to the activation of fast and frugal System 1 thinking rather than systematic System 2 thinking in journalists’ minds.

Leveraging large amounts of digitized collections of text that capture journalists’ actual words produced over an extended time across media, this study draws a more accurate, relevant, and comprehensive picture of how journalists operate in the contemporary media landscape. To date, scholarly efforts that have examined linguistic differences in journalistic outputs are largely focused on qualitative investigation of social hierarchy manifested in journalists’ use of language in a small number of news media reports [30–33, 39] and register variation across media in terms of lexicon-grammatical characteristics of the language [48–50]. While these studies contribute significantly to our understanding of journalists’ word choices, there are gaps in our understanding of how journalists might vary word choices across different media and what the differences in language use reveal about journalists’ cognition. This study bridges this gap by applying cognitive psychological insights to analyze an extensive number of journalistic outputs generated across media over an extended time period. The scope of our analysis facilitates a broader comparison of the way coverage varies across media for reporters covering presidential politics.

### Limitations and directions for future research

Some limitations should be noted when interpreting the present findings. Because we focused on a sample of 73 political journalists [21], this restricts the generality of the conclusions we can draw. Although considerable variance exists in our sample in terms of the reporters’ experience in journalism, partisanship, age, gender, and type of platforms they are mainly affiliated with, future research should incorporate more data from a wider range of journalists covering different domains such as finance, international affairs, and environment.

Another limitation comes from the restricted features in our data. Due to the limited information available at the time of data collection, we could not incorporate potentially relevant variables such as local posting time of tweets. Considering that diurnal variation can relate to sentiment on social media platforms [67], future investigations will need to combine such metadata for more contextualized understanding of the psychology of language. In addition, analysis was drawn from a total of 13,123,538 words collected from papers (*n* = 9,745,292 words), broadcast transcripts (*n* = 237,583 words), and Twitter (*n* = 2,643,593 words). Compared to prior research that focused on a small number of reports from a few, selected mainstream news media [32, 33, 39], relatively large amounts of words were collected and then standardized through the LIWC software [55, 60–62] during the analysis. To the best of our knowledge, this process yields robust findings across media and between individual news reports within each media category, but future investigations need to investigate further if variance in sample size affects results and examine other types of social media journalists often engage with such as Facebook.

Concerns can be raised about the automated text analysis program employed in this study. We used the LIWC dictionaries [55–58] to operationalize journalists’ cognitive mindsets in their writing or spoken words across media. One of the limitations of LIWC is that it uses a bag-of-words approach, meaning it counts words in its pre-defined categories and does not consider contextual clues such as figurative or satirical use of words. Given the large text corpus we analyzed, this off-the-shelf text analysis approach may be an acceptable trade-off [68] but future research should understand how these measurement limitations influence results and explore other text analysis methods that allow less restrictive categorization of words such as unsupervised topic modeling [69].

Lastly, this study focused on the fast-paced setting and informal culture of Twitter and showed how use of different language could be related to journalists’ cognitive mindsets across media. While various dimensions of journalists’ word choices indicate journalists are likely to choose words in line with System 1 thinking on Twitter, this observational study did not directly measure journalists’ perception and motivations for engaging with different media. A recent study [70] analyzed survey responses of journalists’ perception of time pressure from 63 countries. Findings suggest that journalists’ subjective perceptions of time pressure can vary between countries and across media. For example, journalists in developed countries with advanced technology reported higher levels of time pressure. Reporters working for news magazines indicated higher levels of time pressure than peers working for other types of media in part because they used to have a relatively slower publication cycle but now they are increasingly asked to adapt to more accelerated publication cycles through digitized channels. Future research will need to triangulate journalists’ self-reported perceptions of time pressure, motivations of using various media, and how they actually write on these media to understand more fully the differences between their language on Twitter versus in traditional media.

## Conclusion

This study uses cognitive theories to examine how reporters’ language choices can vary across media, with an emphasis on how time pressures affect tweeting by reporters. We construct a novel dataset to analyze the tweets, news articles, and broadcast comments by 73 reporters covering the 2016 presidential campaign. Our findings show how theories of the mind and language can explain levels of emotion, informal language, analytical thinking, and references to the past as reporters cover presidential politics in traditional media and on social media. This study extends the use of cognitive biases in politics to a new realm, reporting, and shows how journalists’ use of language on the campaign trail reflects cognitive biases that arise when individuals make decisions under time pressure and uncertainty.
